# Combinatorial growth of Si nanoribbons

**DOI:** 10.1186/1556-276X-6-476

**Published:** 2011-07-27

**Authors:** Tae-Eon Park, Ki-Young Lee, Ilsoo Kim, Joonyeon Chang, Peter Voorhees, Heon-Jin Choi

**Affiliations:** 1 Department of Materials Science and Engineering, Yonsei University, Seoul 120-749, South Korea; 2 Spin Device Research Center, Korea Institute of Science and Technology, Seoul 136-791, South Korea; 3 Department of Materials Science and Engineering, Northwestern University, Evanston, Illinois 60208, USA

## Abstract

Silicon nanoribbons (Si NRs) with a thickness of about 30 nm and a width up to a few micrometers were synthesized. Systematic observations indicate that Si NRs evolve via the following sequences: the growth of basal nanowires assisted with a Pt catalyst by a vapor-liquid-solid (VLS) mechanism, followed by the formation of saw-like edges on the basal nanowires and the planar filling of those edges by a vapor-solid (VS) mechanism. Si NRs have twins along the longitudinal < 110 > growth of the basal nanowires that also extend in < 112 > direction to edge of NRs. These twins appear to drive the lateral growth by a reentrant twin mechanism. These twins also create a mirror-like crystallographic configuration in the anisotropic surface energy state and appear to further drive lateral saw-like edge growth in the < 112 > direction. These outcomes indicate that the Si NRs are grown by a combination of the two mechanisms of a Pt-catalyst-assisted VLS mechanism for longitudinal growth and a twin-assisted VS mechanism for lateral growth.

## Introduction

One-dimensional nanostructures have attracted much attention in the research community owing to their novel physical and chemical properties and due to their easy manipulation as building blocks for nanoscale devices. In particular, nanoribbons (NRs) are of interest on account of their geometrical shape, comprised of a rectangular cross-section on a nanometer scale that can provide unique properties for optical, mechanical, and electrical devices. Limited experiments on III-V and oxide semiconductor NRs have already shown promising properties, such as the wave-guiding of photons, lasing action, nonlinear polarization, Aharonov-Bohm interference, and high mechanical flexibility [1-5]. Meanwhile, it is highly advantageous for device application if the NRs can be fabricated with a semiconductor compatible with the complementary metal-oxide semiconductor process. A good example here is a semiconductor made of silicon (Si).

Two different methods to prepare Si NRs have been reported. The top-down approach uses lithography and etching procedures to create the NRs from wafers, which affords a well-defined morphology and crystalline orientation [6]. Meanwhile, the bottom-up approach uses chemical synthesis with molecular precursors to synthesize the NRs by an oxide-assisted growth (OAG) or vapor-liquid-solid (VLS) mechanism [7,8]. However, the fabrication of Si NRs via the bottom-up approach is still in its nascent stage; developing reliable synthesis processes as well as understanding the growth mechanism are crucial to exploit the potential of Si NRs. Herein, we report the synthesis of Si NRs and their combinatorial growth mechanism consisting of a metal-catalyst-driven VLS and a defect-driven vapor-solid (VS) mechanism.

### Experimental procedure

Si NRs were synthesized on Si (111) substrates using CVD process. Conventional wet chemical cleaning processes were performed to remove any residual components from the substrates. Pt thin film (0.5 nm) was deposited as catalyst by using the electron-beam evaporator. The substrates were then placed in a hot-wall horizontal reactor and heated to the reaction temperature of 1,000°C under a H_2 _(99.9999%) and an Ar (99.9999%) flow of 100 and 100 standard cubic centimeter per min (sccm), respectively. SiCl_4 _(Aldrich, 99.9999%, Aldrich Chemical Co., Milwaukee, WI, USA) was then supplied for 10 min by bubbling with H_2 _as a carrier gas at 20 sccm. The carrier gas was then turned off and the reactor was cooled to room temperature.

The structural properties of the Si NRs were characterized using scanning electron microscopy (SEM) (Hitachi 3000, Hitachi Co., Tokyo, Japan) and transmission electron microscopy (TEM) (JEOL 7100, 200 keV, JEOL, Tokyo, Japan). To prepare the samples for TEM observation, the NRs were dispersed via the ethanol solution. A small droplet of the solution was then dropped onto the copper TEM grid. To prepare the samples for cross-section TEM observation, the saw-like edged NRs were dispersed via ethanol solution onto Ge substrates coated with 30 nm of Au film. The cross-sectional samples were produced by FIB (Nova 600 Nanolab) and a lift-out technique (Figure S1 in Additional file 1). The cross-sectional samples were affixed to TEM grid and sliced to electron transparency with progressively smaller ion-beam currents.

## Results and discussion

**Figure 1:**
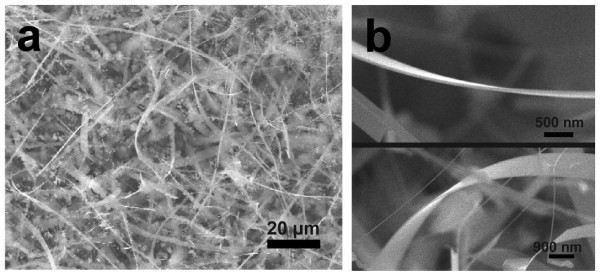
**SEM image of Si NRs**. (**a**) Typical SEM image of Si NRs grown on a Si substrate. (**b**) SEM images of an individual Si NR.

Si NRs were synthesized on Si substrates assisted by Pt as a catalyst via chemical vapor transport system [9,10]. Figure 1a shows a SEM image, showing a large quantity of flexible Si NRs on the substrate. Most of the NRs have a thickness between 30 and 40 nm, a width of a few micrometers, and a length of a hundreds of micrometers (Figure 1a, b).

**Figure 2:**
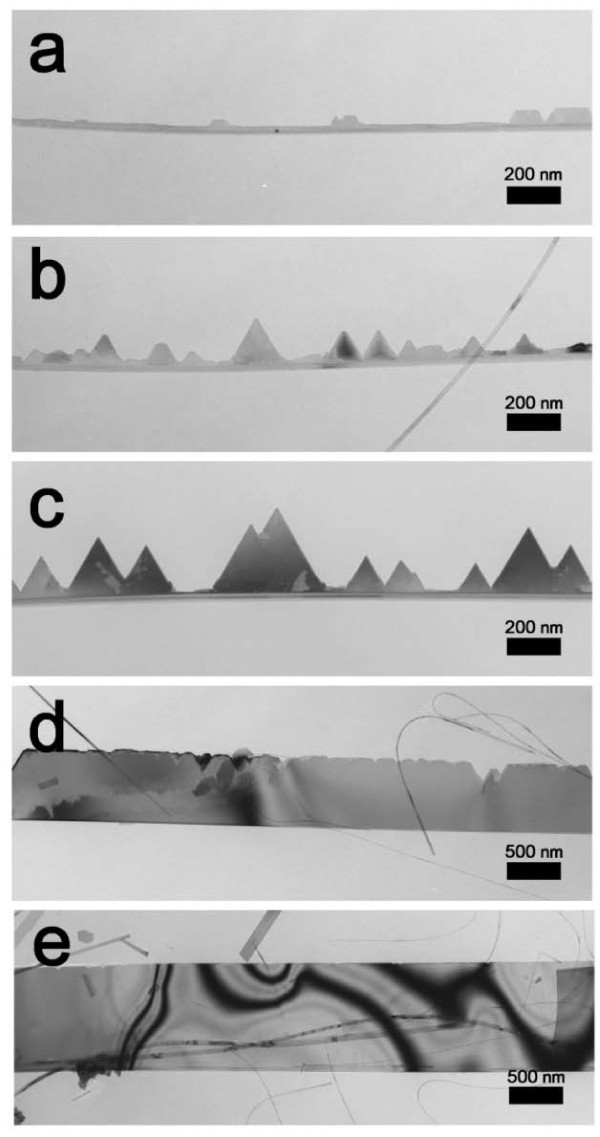
**TEM images of NR**. (**a**-**e**) TEM images showing the evolutionary stages of the NR; basal nanowire, saw-like edges on the basal nanowire, and the NR.

To address the growth mechanism, the evolution of Si NRs over time was examined by TEM. While the degree of evolution differed from ribbon to ribbon, a general trend could be acknowledged. Figure 2a-e shows the typical sequential evolution of the NRs with a processing time interval of 2 min. Initially, Si basal nanowires grew, as shown in Figure 2a. Subsequently, the saw-like edges began to grow along the basal nanowires (Figure 2b-d), the interspaces between the saw-like edges filled, and eventually the NRs shown in Figure 2e resulted. Our observation indicated that the triangular islands are distributed along a ribbon uniformly, as shown in Figure 2b, c. Meanwhile, the average number of islands that is estimated from 15 ribbons is 9 ± 3/μm. These indicate that the distribution of islands in a single ribbon is rather uniform; however, is not quite uniform among different ribbons under same synthesis conditions.

**Figure 3:**
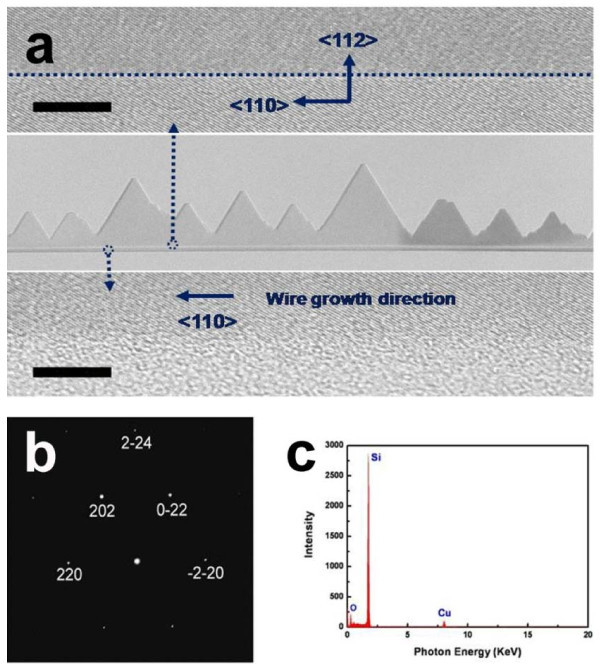
**HRTEM images of NRs**. (**a**) HRTEM images showing the crystallographic orientation of the nanowire with saw-like edges in the course of the conversion to the NRs. The inset at the top shows interface between the basal nanowire and saw-like edge. The inset at the bottom shows the basal nanowire. The scale bar in the images is 5 nm. Corresponding SAED pattern recorded along the [-111] zone axis (**b**) and EDS spectrum (**c**).

To understand the crystal structure of the NRs, the saw-liked NRs were investigated by TEM, as shown in Figure 3. The selected-area electron diffraction (SAED) pattern recorded along [-111] zone axis (Figure 3b) indicated that the basal nanowires within the NRs grew along the < 110 > direction, whereas the saw-like edges grew along the < 112 > direction. As shown in the inset at the top of Figure 3a, no grain boundaries, misfit dislocations, or abrupt interfaces were observed at the interface between the basal nanowire and the saw-like edges. This indicates that the saw-like edges have an epitaxial relationship with the basal nanowires. The energy-dispersive spectroscopy (EDS) analysis presented in Figure 3c shows that the NRs is free from impurities, including Pt.

**Figure 4:**
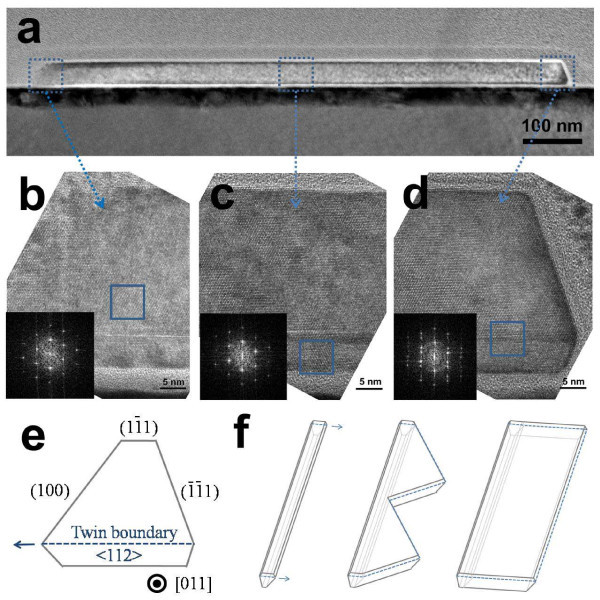
**Cross-sectional TEM and HRTEM images of NR**. (**a**) Cross-sectional TEM image of the saw-like edged NR. (**b**-**d**) Cross-sectional HRTEM images of the three regions (the end part of the saw-like edge, the middle part of the saw-like edge, and the part of the basal nanowire) indicated in panel (a). The insets of (b-d) show diffractograms of the Si region in the box in each part. These indicate that the basal nanowire was grown along < 110 > direction and that the Si nanosaw/NR is bi-crystalline containing a single {111} twin. (**e**) Schematic diagram of the projected shape and facets of the basal nanowire part. (**f**) Schematic showing the formation of the Si NR.

To investigate the structure of NRs in detail, cross-sectional samples of the saw-like edged NRs were prepared by focused ion beam (FIB) slicing and a lift-out process with a micromanipulator (Figure S1 in Additional file 1). This was then observed by TEM. Figure 4a shows a TEM cross-section image of the as-grown Si NRs. The right side of the TEM image in Figure 4a is the part of the basal nanowire, whereas the other side is the part of the saw-like edge. The width of the saw is approximately 1 μm, and its thickness is about 35 nm, as shown in Figure 1b and 4a-d. Further scrutiny of the morphology of the cross-sectional NRs shows no distinct interfaces, which confirms the epitaxial relationship between the basal nanowire and the saw-like edges. Figure 4b-d show cross-sectional high-resolution transmission electron microscopy (HRTEM) images of the NRs, indicating that the basal nanowires have hexagonal cross-sections. Indeed, < 110 > -oriented Si nanowires have been also shown to have hexagonal cross-sections [11,12].

It was interesting to note that the twin extending in the lateral growth direction of the basal nanowires is oriented parallel to the < 112 > direction, as shown in Figure 4b-d. The insets of Figure 4b-d show the fast Fourier transform (FFT) of the corresponding HRTEM images. The FFT diffractogram in the inset of Figure 4d shows that Si NRs is bi-crystalline, containing a single {111} twin. The growth direction of the basal nanowire is along < 110 > direction. According to the TEM outcome, the structure of the basal nanowire can be depicted as shown in Figure 4e, where the < 110 > -oriented the basal nanowire exhibits a hexagonal cross-section bounded by four {111} facets and two {100} facets with a single {111} twin. This twin boundary extends along the < 112 > direction, which corresponds with the lateral growth direction of the NRs.

Based on these results, the growth mechanism of Si NRs can be described as follows. First, relatively thin Si nanowire with a diameter of 30 nm grows on the Si substrate assisted by Pt as a VLS catalyst (Figure 2a). Our previous study of nanowires from the initial stage showed Pt catalyst at the end of many nanowires [9]. The basal nanowires were grown in the < 110 > direction. This result stems from the interplay of the liquid-solid interfacial energy with the Si surface energy expressed in terms of the edge tension in this diameter regime of 30 nm [13-15]. The basal nanowires have twins that extend to the side edges. The formation of twins in the nanowires has also been reported with Si or Ge nanowires grown in the < 112 > and < 110 > directions by the VLS or a supercritical fluid-liquid-solid (SFLS) mechanism [16-19]. Twin formation in these cases occurs during nanowire nucleation and it extends down the length of the nanowires as the nanowires grow because the twins can provide preferential addition sites that maintain nanowire growth in the energetically favorable < 112 > or < 110 > direction.

It is noted that Pt catalysts have not been found in the NRs. This may occur due to the etching out of Pt-Si liquid globules during the course of growth under a chloride atmosphere. Because the chemical activity of the liquid metal globules becomes higher as the diameter becomes smaller according to the Gibbs-Thompson effect, Pt-Si liquid globules with a diameter of around 30 nm could be etched out after the initial stage under chemically harsh conditions.

The twin appears to play an important role in the subsequent lateral growth (i.e., the growth of the saw-like edges) from the basal nanowires by the VS mechanism. In fact, previous studies suggest that twins have critical roles in the crystal growth. For example, the presence of a twin can drive the growth in a specific direction by what is known as classical "reentrant twin mechanism" [20,21]. Indeed, the reentrant twin mechanism has already been suggested for the growth of Si ribbons, which are very similar morphology to our Si NRs though the size were much bigger (width of 30-150 μm and length of 1-20 mm) [20]. Here, {111} twin creates favorable nucleation sites at the growth interfaces and atoms arriving from the vapor phase can readily accommodated at the nucleation sites, which will drive a rapid net growth in the < 112 > direction.

Regarding the role of the twin on the lateral growth, it is also noted that the twin creates distinct surface energy anisotropy in the basal nanowire. As shown in Figure 4e, the twinned Si nanowires have mirror-like crystal structures in which the two {100} planes are adjacent on one side while the other four facets consisted of {111} planes. The surface energy of the {100} facet is higher than that of the {111} facet [22]; thus, such a mirror-like crystallographic configuration results in anisotropic surface energy states in a specific direction (i.e., the < 112 > direction). This type of anisotropic surface energy can also induce preferred crystal growth at surfaces where the surface energy is high (i.e., the direction of two {100} facets in the basal nanowires) to minimize the surface energy associated with high-energy facets. Therefore, besides reentrant mechanism, the twin could further drive lateral growth from the basal nanowires by the VS mechanism by creating an asymmetric crystallographic configuration and thus an asymmetric surface energy state. As mentioned earlier, Pt or other types of impurities were not found in the saw-like edges or NRs. Hence, this lateral growth would occur without the assistance of a metal catalyst.

The triangular configurations of the saw-like edges are due to the nucleation of two-dimensional islands during the epitaxial growth on the Si (111) surface [23,24]. On the Si (111) surface, a triangular island can be formed by the slow growth rate of two low-index step edge facets ([1-12] and [11,12]) inducing the formation of the triangular island. The subsequent process of the filling of the saw-like edges may be due to anisotropic growth kinetics. As described, the < 112 > directions are the fast growth directions. The sides of the triangles then move quicker than the other orientation. The triangles form and < 112 > -oriented facets grow out of the system leaving the {100} planar surface. In this case, the width of the ribbon would be related to the density of the island nucleation sites where large triangles will form when there are a few nucleation sites and the width of the ribbon would be equal to the height of the largest triangle before it gets in contact with another triangle. When the density of triangular islands is high, the width of the ribbon would be smaller.

Figure 4f shows a schematic diagram that summarizes the evolution of Si NRs. As shown here, the nanowires grow first along the < 110 > direction with a single {111} twin via the VLS mechanism with a Pt catalyst. The saw-like edges then grow from the side of the nanowire along the < 112 > direction via the twin-driven VS mechanism with further filling of the edges by the selective condensation of vapor driven by the chemical potential differences. Recently, free-standing Si nanosheets with a thickness of about < 2 nm has been reported using similar synthesis conditions [25]. The difference between the nanosheets and nanoribbons reported here is growth mechanism, wherein the former is grown by VS mechanism without catalyst while the latter is grown by combinatorial VLS and VS mechanism using metal catalyst. By considering the potential of catalyst and VLS mechanism for the control of morphology of Si nanostructures, the combinatorial mechanism reported here may be helpful to create versatile one-dimensional Si nanostructures.

## Conclusion

The bulk of previous studies have reported the growth of one-dimensional Si nanostructures (i.e., nanowires and NRs) via the VLS or the VS mechanism, respectively [8,15,26,27]. Our study implies that a combination of these two well-established growth mechanisms makes it possible to prepare novel Si nanostructures such as Si NRs that can be used for optical, mechanical, and electrical devices. Although the combinatorial approach in this study only showed the growth of Si NRs, the concept of this approach can be applied as a reliable process to prepare many other novel one-dimensional nanostructures.

## Competing interests

The authors declare that they have no competing interests.

## Authors' contributions

TEP carried out the main part of synthesis, the structural analysis, and drafted the manuscript. KYL and IK participated in the structural analysis. JC participated in the discussion of the cross-sectional TEM sampling. PV participated in the discussion of the growth mechanism. HJC participated in the design of the study, draft preparation and coordination. All authors read and approved the final version of the manuscript.

## Supplementary Material

Additional file 1 **Combinatorial Growth of Si Nanoribbons**. Supporting Information Click here for file
