# Supplementary material for: Combinatorial growth of Si nanoribbons
Source: Nanoscale Res Lett. 2011 Jul 27;6(1):476. doi: 10.1186/1556-276X-6-476 (PMC3211989; doi:10.1186/1556-276X-6-476)
Supplement: Additional file 1 — Combinatorial Growth of Si Nanoribbons. Supporting Information [file 1556-276X-6-476-S1.DOC]

**Supporting Information**

**Combinatorial Growth of Si Nanoribbons**

Tae-Eon Park,^1,2^ Ki-Young Lee,^1^ Il-Soo Kim,^1^ Joonyeon Chang,^2^ Peter Voorhees,^3^ and Heon-Jin Choi^*,1^

^1^ Department of Materials Science and Engineering, Yonsei University, Seoul, Korea

^2^Nano Convergence Device Center, Korea Institute of Science and Technology, Seoul, Korea

^3^ Department of Materials Science and Engineering, Northwestern University, United States

Science and Technology, Seoul, Korea and Advanced Analysis Center, Korea Institute of Science and Technology, Seoul, Korea, and Department of Physics, Yonsei University, Seoul, Korea

1. **Methods of cross-sectional TEM samples**
   1. Synthesis of Si saw-like edged NRs

Si saw-like edged NRs were synthesized using Si substrates with the assistance of Pt as catalyst under flows of argon and hydrogen at 1000℃for 6 min. in a horizontal hot-wall chemical vapor transport system (Figure S1a). The saw-like edged NRs as cross0section TEM samples were selected because for this state, it is easy to discern the basal nanowire site and the saw-like edge site.

- 1. Fabrication of cross-sectional TEM samples

To fabricate the cross-sectional TEM samples, Si saw-like edged NRs were dispersed via ethanol solution onto Ge substrates coated with 30 nm of Au, as shown in Figure S1b. Recently, fabrication of the cross-sectional TEM samples of nanostructures has been done by a slicing followed by a lift-out process with a micromanipulator.^S1,S2^ First, the region of interest was selected, upon which a protective Pt layer was deposited using an electron beam (Figure S1c). This layer was provided to protect the underlying sample from ion-beam damage during the milling process. Next, a Pt strap was deposition over the region of interest (Figure S1d) to mill out a trench on either side of the Pt region. This region was then gripped with FIB micromanipulator controls and cur free from the substrate. Moving the slab to the TEM grid involved affixing the micromanipulator probe via the deposition of Pt and cutting it free from the micromanipulator tip, as shown in Figure S1e. The sample was then sliced to electron transparency via ion milling. The right side of the SEM image in Figure S1f is the basal nanowire site, whereas the other side is the saw-like edge site.


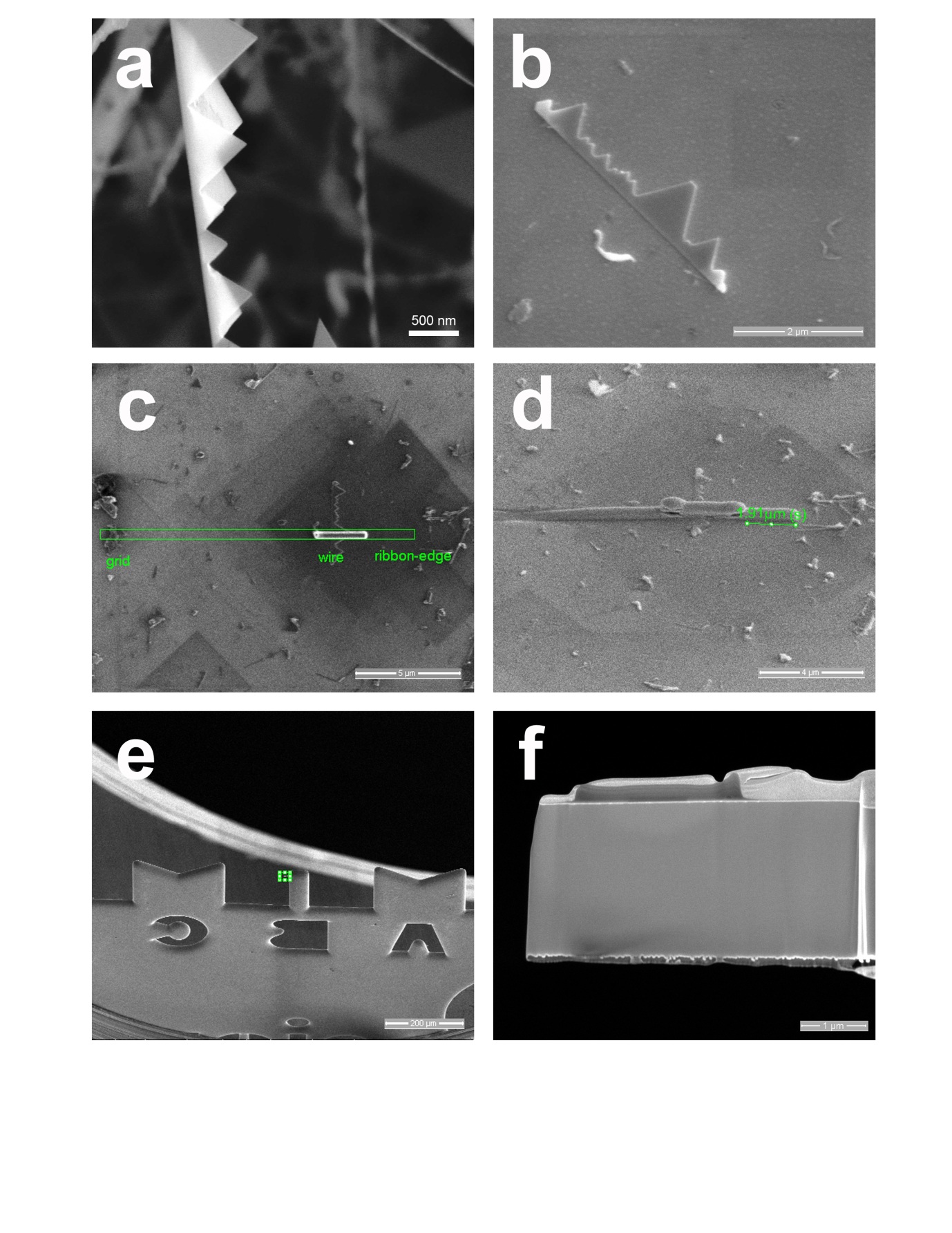


**Figure S1.** SEM images illustrating the steps in the FIB slicing and lift-out with a micromanipulator to prepare the cross-sectional TEM samples. (a) Si saw-like edged NRs grown on Si substrates. (b) Each saw-like edged NR was distributed on a Ge substrate coated with an Au layer. (c) The region of interest of the saw-like edged NR was selected, and a Pt layer was deposited using an electron beam. (d) A Pt strap was deposited over the area of interest to mill out the trench. (e) The sample was then transferred to the TEM grid and attached to it with a micromanipulator. (f) Side view of the cross-sectional saw-like edged NR sample after slicing.

**REFERENCES**

S1. Tham, D.; Nam, C. Y.; Fischer, J. E. Adv. Funct. Mater. **16**, 1197 (2006).

S2. Liu, Z.; Elbert, D.; Chien, C. L.; Searson, P. C. Nano Lett. **8**, 2166 (2008).
